# Nutritional Vulnerability and Functional Decline in End-Stage Heart Failure and Chronic Respiratory Disease: Utility of the CONUT Score in a Palliative Cohort

**DOI:** 10.3390/nu17193040

**Published:** 2025-09-24

**Authors:** Martina Pellicé, Andrea Ladino, Karla Belén Treviño-García, Ana Suárez-Lombraña, Marta Arroyo-Huidobro, Aina Capdevila-Reniu, Bryan David Solari, Emilio Sacanella, Juan Manuel Perez-Castejon, Ferran Masanes

**Affiliations:** 1 Geriatric Department, Hospital Clinic of Barcelona, School of Medicine, University of Barcelona, Villarroel Street 170, 08036 Barcelona, Spain; ladino@clinic.cat (A.L.); arroyo@clinic.cat (M.A.-H.); aicapdev@clinic.cat (A.C.-R.); bdsolari@clinic.cat (B.D.S.); fmasanes@clinic.cat (F.M.); 2 Internal Medicine Department, University Hospital Dr. Jose Eleuterio González, Universidad Autónoma de Nuevo León, Monterrey 64460, Mexico; karla93_@hotmail.com; 3 Internal Medicine Department, Hospital Clinic of Barcelona, School of Medicine, University of Barcelona, 08036 Barcelona, Spain; ansuarez@clinic.cat (A.S.-L.); esacane@clinic.cat (E.S.)

**Keywords:** malnutrition, CONUT, palliative care, chronic heart failure, chronic respiratory disease, nutritional assessment, end-stage diseases

## Abstract

Background/Objectives: Malnutrition is common among patients with advanced chronic illnesses receiving palliative care, yet comparative data between diagnostic groups are limited. This study aimed to evaluate and compare the nutritional status of patients with end-stage chronic heart failure (CHF) and chronic respiratory disease (CRD), and to assess the clinical utility of the Controlling Nutritional Status (CONUT) score in this setting. Methods: We conducted a retrospective analysis of 80 patients (41 with CHF, 39 with CRD) enrolled in a palliative care program (mean age 77.8 ± 6.8 years, 65% male). Nutritional status was assessed using BMI (Body Mass Index), CONUT score, and routine biochemical markers. Functional and clinical variables, including the Palliative Performance Scale (PPS), were also collected. Results: Moderate-to-severe malnutrition (CONUT ≥ 5) was significantly more prevalent in patients with CHF patients (44%) than CRD patients (10%, *p* = 0.002). CHF patients exhibited lower BMI, cholesterol, lymphocyte counts, and prealbumin levels. Despite more frequent nutritional follow-up and protein supplementation in the CHF group, these interventions were not associated with improved nutritional classification. The CONUT score correlated more strongly with functional impairment (PPS) than with disease type alone. Conclusions: Patients with CHF receiving palliative care demonstrate higher rates of malnutrition than those with CRD. The CONUT score, derived from standard blood test, may be pragmatic screening tool for identifying nutritional vulnerability and guiding interventions. While it does not predict survival, it may help detect functional decline earlier and support care strategies aimed at maintaining quality of life in end-stage disease.

## 1. Introduction

In advanced chronic disease such as end-stage heart failure (HF) and chronic respiratory disease (CRD), palliative care prioritizes symptom management, functional preservation, quality of life, and clinical decision-making aligned with patient goals [1,2,3]. In this context, nutritional status is not only a consequence of disease progression, but also a potentially modifiable factor that affects quality of life, clinical outcomes and decision-making [4,5]. Despite this, the clinical integration of nutritional assessment into palliative pathways remains limited and inconsistent.

Malnutrition and cachexia are common in both HF and CRD, driven by a combination of systemic inflammation, metabolic alterations, and reduced intake [6]. In HF, according to the literature, between 30% and 60% of patients develop malnutrition, which is associated with increased mortality, hospitalization, and frailty [7,8,9,10,11]. In CRD, nutritional deterioration also correlates with muscle wasting, lower respiratory function, and poorer prognosis [12]. Sarcopenia and low BMI correlate with worse pulmonary function and higher mortality [13]. Nutritional support in these settings can improve dyspnea, strength, and quality of life [14]. However, the underlying inflammatory state and frequent comorbidities pose challenges to standard nutritional classification and intervention. Nutritional support, including individualized counseling and protein supplementation, has shown benefits in both HF and CRD, such as improved function, symptom control, and possibly reduced hospitalizations [15,16]. However, evidence in palliative contexts is scarce, and clinical decision-making often relies on fragmented information. The optimal timing, indications and outcomes of nutritional interventions in patients near end of life remain ill-defined.

Traditional tools such as body mass index (BMI) fail to adequately reflect nutritional deficits in this population, as they do not account for fluid overload, muscle loss, or inflammatory states [17]. The CONUT score, based on serum albumin, total cholesterol, and lymphocyte count, has emerged as a pragmatic alternative [18,19,20,21,22,23]. While it offers promise for routine screening, its interpretation in multimorbid, inflamed, and end-stage patients remains uncertain, given the influence of non-nutritional factors on its components. However, its components are influenced by non-nutritional factors such as inflammation and renal dysfunction, which are prevalent in end-stage HF and CRD, potentially limiting its specificity [22]. Compared to GLIM criteria, which incorporate muscle mass and etiology [6], or the Prognostic Nutritional Index (PNI) [22], CONUT offers simplicity but may require cautious interpretation in palliative settings.

Despite the existence of several nutritional screening tools, the choice of the CONUT score in this study was driven by its simplicity, reliance on routine laboratory data, and its potential applicability in real-life clinical settings where time and resources are limited. However, its performance in end-stage non-oncological palliative populations remains understudied and potentially confounded by inflammation and comorbidities.

We focused on patients with end-stage HF and CRD because they represented the most numerous non-oncological palliative subgroups referred to our unit. Both conditions are highly prevalent and associated with malnutrition and functional decline, yet comparative data remain scarce. Therefore, this study aimed to compare the nutritional status of patients with end-stage HF and CRD receiving palliative care, to identify clinical predictors of malnutrition, and to evaluate the potential utility of CONUT in this setting. Beyond this comparison, the study also assessed the CONUT score as a pragmatic screening tool for nutritional vulnerability in palliative patients with chronic heart or respiratory diseases.

## 2. Materials and Methods

### 2.1. Design, Setting and Participants

This retrospective cross-sectional study analyzed nutritional data collected during routine clinical assessments of patients with end-stage heart failure (HF) and chronic respiratory disease (CRD) enrolled in a non-oncological palliative care program at a tertiary-level hospital in Barcelona, Spain. The hospital serves as a referral center for complex chronic conditions. Data were collected from August 2022 to March 2025, with survival follow-up until May 2025. Patients with a confirmed terminal diagnosis by cardiology or pulmonology specialists were included, with no specific exclusion criteria beyond the absence of a terminal diagnosis.

### 2.2. Data Collection

Demographic characteristics (e.g., age, sex and origin) and clinical characteristics (e.g., dementia and type of disease) were obtained from electronic medical records. The palliative care team assessed functional status using the Barthel Index, the Clinical Frailty Scale (CFS), and the Palliative Performance Scale (PPS). The NECPAL CCOMS-ICO tool was used to stratify palliative care needs. Cardiac data (disease type, LVEF (Left Ventricular Ejection Fraction)) and NYHA (New York Heart Association) and respiratory data including disease type and severity, assessed using the modified Medical Research Council (mMRC) dyspnea scale, were assessed using standardized interviews. Body mass index (BMI) was included as a routinely used anthropometric parameter to allow comparability with previous studies, despite its limitations as a marker of malnutrition. In addition, the CONUT score (Controlling Nutritional Status), calculated from biochemical markers such as albumin, cholesterol and lymphocytes count, as well as data from interviews on dietary intake and supplementation, were collected from the medical records based on nutritionists’ assessment. However, nutritional follow-up and supplementation were not protocolized and may have varied based on clinical judgment. Biochemical markers (total protein, albumin, prealbumin, etc.) were obtained from routine blood tests.

For the secondary survival analysis, data on mortality were collected until 30 May 2025, through retrospective review of clinical records and death notifications, with the date of initial palliative care assessment defined as the start of follow-up.

### 2.3. Study Variables

We also collected data on weight loss in the previous 6 months, whether there was follow-up by a nutritionist, protein supplement intake, and adherence. Nutritional biochemical markers included serum protein (total protein, albumin, prealbumin), lipid profile (total cholesterol, triglycerides), minerals (phosphorus, magnesium, zinc, calcium), renal marker (creatine), immune marker (lymphocyte count), and C-reactive protein (CRP). Biochemical parameters were determined in the central laboratory using standard automated methods: serum proteins by colorimetric assay, albumin by bromocresol green, prealbumin by immunoturbidimetry, cholesterol and triglycerides by enzymatic colorimetry, phosphorus, magnesium, zinc, and calcium by spectrophotometry, creatinine by Jaffe method, lymphocyte count by automated hematology analyzed, and CRP by high-sensitivity immunoturbidimetry.

The other variable were age (years), sex (male/female), origin (home, residence, or intermediate center), dementia (yes/no), dependency level (Barthel Index: Independent, mildly dependent, moderately dependent, severely dependent), frailty level (CFS 1–9), palliative care needs (NECPAL I–III), and palliative performance level (PPS > 60, 30–50, or 10–20). The type of heart disease (Ischemic, Idiopathic/Familial Dilated, Hypertrophic, Valvular, Amyloidosis, Transplant or Ischemic-valvular) and respiratory disease (Chronic Obstructive Pulmonary Disease (COPD), Diffuse Interstitial Lung Disease (ILD), Pulmonary Hypertension (PH) or other) was recorded. The stage of the disease was assessed using LVEF (normal [>50–55%], mildly [41–49%], reduced [≤40%], or severely reduced [<30%]), the NYHA (class I–IV), cardiac treatments (inotropic (Levosimendan or Dobutamine), Hemodialysis (peritoneal or conventional), inotropes and peritoneal dialysis, diuretic or external ventricular drain), and respiratory treatments (home oxygen, home high-flow nasal cannula, nocturnal CPAP or bronchodilator therapy). Symptoms included dyspnea (no dyspnea, at rest or in effort), anorexia, asthenia/fatigue, xerostomia, dysphagia, pain and constipation.

The CONUT score was calculated according to the original methodology described by de Ulibarri et al. (2005) [23], based on three parameters: serum albumin concentration, total cholesterol concentration, and lymphocyte count. Each parameter was assigned a score, and the sum classified patients into four categories: normal (0–1), mild (2–4), moderate (5–8), and severe (9–12). For regression analyses, we dichotomized the variable into normal/mild (0–4) versus moderate/severe (≥5), given the very low prevalence of severe cases in our cohort.

For the survival analysis, additional variable was time to death (months from initial assessment to death or study closure) and event status (deceased or censored).

### 2.4. Statistical Analyses

Data were analyzed using IBM SPSS Statistics version 28.0 (IBM Corp., Armonk, NY, USA). Continuous variables were presented as means ± standard deviation (SD) or medians (interquartile range, IQR) according to their distribution, assessed using the Shapiro–Wilk test. Categorical variables were expressed as frequencies and percentages. Differences between HF and CRD groups were assessed using *t*-test or Mann–Whitney U tests for continuous variables (based on Shapiro–Wilk normality test) and Chi-square or Fisher’s exact test (when ≥50% of cells had expected frequencies <5) for categorical variables. CONUT scores were categorized as Normal (0–1), Mild (2–4), Moderate (5–8), and Severe (9–12) for descriptive analysis, and dichotomized (Normal/Mild (0–4) vs. Moderate/Severe (≥5) for regression due to low Severe cases (n = 3). Univariate analyses used Chi-square/Fisher’s exact test for categorical variables (e.g., specialty, NECPAL, PPS, symptoms, nutritional factors) and Mann–Whitney U for continuous variables (e.g., albumin, cholesterol, lymphocytes). Odds ratios (ORs) with 95% CIs were calculated via univariate logistic regression. A complementary ordinal logistic regression was conducted using the original three CONUT categories (Normal, Mild, Moderate/Severe) to account for nutritional severity. Due to the limited sample size and high correlation between functional scales (e.g., Pearson’s r = 0.75 between PPS and Barthel Index, *p* < 0.001), the Palliative Performance Scale (PPS) was retained in the multivariate model as a composite measure of functional capacity and clinical severity, reducing the risk of overparameterization and collinearity with NECPAL and Barthel Index. Survival was analyzed using Kaplan–Meier curves and the log-rank test. Median survival times and 95% CIs were reported for key variables. The impact of nutritional status and other clinical predictors on survival was further assessed using Cox proportional hazards regression, expressed as hazard ratios (HR) with 95% CI. A *p*-value < 0.05 was considered statistically significant.

## 3. Results

### 3.1. Characteristics of the Study Population

A total of 80 palliative care patients were included, with 41 (51%) diagnosed with end-stage heart failure and 39 (49%) diagnosed with end-stage chronic respiratory disease. The mean age was 77.8 years (SD 6.8), with a predominance of males (65%). No significant differences were observed between the two groups in terms of age (*p* = 0.382), sex (*p* = 0.870), origin (home, nursing home, or intermediate care; *p* = 0.751), prevalence of dementia (*p* = 0.616), functional dependence (Barthel Index; *p* = 0.925), or frailty (Clinical Frailty Scale; *p* = 0.663). Palliative care needs (NECPAL) differed significantly, with 51% of cardiac patients classified as NECPAL III compared to 8% of respiratory patients (*p* < 0.001). Overall mortality was 65%, with no significant difference between cardiac (73%) and respiratory (56%) patients (*p* = 0.116) (Table 1).

### 3.2. Clinical and Symptom Profile

Regarding clinical characteristics, cardiology patients most frequently presented with valvular (36%) and ischemic (20%) heart disease, with 61% exhibiting reduced or severely reduced left ventricular ejection fraction (LVEF ≤ 40%) and 64% classified as NYHA class III–IV. In this group, diuretic use was common (56%), and 25% received inotropic support. In contrast, respiratory patients were primarily diagnosed with ILD (49%) and COPD (38%). Most (82%) reported severe dyspnea (mMRC 3), and 74% required home oxygen therapy. Among the total cohort, six patients were receiving dialysis (three on hemodialysis and three on peritoneal dialysis). Due to the very small numbers, no separate analysis was performed.

Symptom profiles also differed: dyspnea at rest or on exertion was more common in the respiratory group (*p* < 0.001) while anorexia was more prevalent in cardiac patients (59% vs. 36%, *p* = 0.043). No statistically significant differences were found for asthenia, xerostomia, dysphagia, pain or constipation (Table 2).

### 3.3. Nutritional Status and Biochemical Markers

Nutritional status, assessed by the Controlling Nutritional Status (CONUT) score, showed significant differences between groups (*p* = 0.001, chi-square test). Among cardiac patients, 44% had moderate/severe malnutrition (CONUT ≥ 5) compared to 10% of respiratory patients. Conversely, 90% of respiratory patients had normal/mild nutritional status (CONUT 0–4) compared to 56% of cardiac patients (Table 3).

Body mass index (BMI) was lower in cardiac patients (median 22.65, IQR 5.36) than in respiratory patients (median 25.13, IQR 8.87; *p* = 0.045, Mann–Whitney U test). Nutritional follow-up was more prevalent in cardiac patients (66% vs. 44%, *p* = 0.045). This may reflect a perceived higher nutritional risk in this group, rather than protocol-driven intervention. No significant differences were observed in weight loss in the past 6 months (27% vs. 18%, *p* = 0.342), protein supplementation (49% vs. 36%, *p* = 0.244), or adherence to supplementation (100% vs. 85%, *p* = 0.251).

BMI showed a weak but statistically significant inverse correlation with CONUT (Spearman’s *p* = −0.226, *p* = 0.044; Pearson r = −0.221, *p* = 0.049, suggesting that higher BMI was only marginally associated with lower nutritional risk.

Biochemical markers also differed significantly. Cardiac patients had higher total protein levels (65.98 ± 8.85 g/L vs. 59.51 ± 7.77 g/L, *p* = 0.001), prealbumin (0.21 ± 0.07 g/L vs. 0.15 ± 0.07 g/L, *p* = 0.006), phosphorus (median 3.75, IQR 1.1 vs. 3.20, IQR 0.6 mg/dL, *p* = 0.005), calcium (median 9.00, IQR 0.8 vs. 8.60, IQR 0.5 mg/dL, *p* = 0.019), and creatinine (median 2.05, IQR 1.75 vs. 0.81, IQR 0.25 mg/dL, *p* = 0.001). Conversely, respiratory patients had higher total cholesterol (median 175.00, IQR 68 vs. 125.00, IQR 59 mg/dL, *p* < 0.001) and lymphocyte counts (median 1.70, IQR 1.10 vs. 0.82, IQR 0.80 × 10^9^/L, *p* < 0.001). No significant differences were found in albumin, triglycerides, magnesium, zinc, or C-reactive protein (Table 3).

### 3.4. Predictors of Malnutrition

A univariate analysis was performed to identify predictors of moderate/severe malnutrition defined as (CONUT ≥ 5). Cardiac specialty was a significant predictor (OR = 6.85; 95% CI: 2.06–22.78; *p* = 0.001), NECPAL stage III (*p* = 0.013), PPS < 50 (*p* = 0.001), low Barthel Index (*p* = 0.007), nutritional follow-up (OR = 3.91; 95% CI: 1.29–11.86; *p* = 0.022), protein supplementation showed a trend toward statistical significance (OR = 2.55 (0.93–6.96); *p* = 0.080. Several biochemical parameters (low albumin, low cholesterol, low lymphocytes, and elevated creatinine; all *p* < 0.05). Sex, age, BMI category, LVEF, weight loss and presence of symptoms (e.g., anorexia, dysphagia, constipation, xerostomy) were not significantly associated (Table 4).

To identify independent predictors of nutritional impairment in the study population, two multivariate regression models were performed using the CONUT score as an outcome variable.

#### 3.4.1. Binary Logistic Regression for Moderate/Severe Malnutrition (CONUT ≥ 5)

A binary logistic regression model was constructed to assess factors associated with (CONUT ≥ 5). Predictor variables were selected based on their statistical significance (*p* < 0.05) in the univariate analysis (Table 4), excluding biochemical components already incorporated within the CONUT score to avoid collinearity. Initially, both the Barthel Index and NECPAL stage were included. However, the model was overparameterized due to the limited sample size. Preliminary analyses showed moderate-to-high correlations between PPS, Barthel Index (r = 0.75, *p* < 0.001), and NECPAL stage (r = 0.62, *p* < 0.001), justifying the inclusion of PPS as a proxy for functional and clinical severity in the multivariate model to avoid overparameterization. The final model demonstrated a good fit (Hosmer–Lemeshow test, *p* = 0.903) and explained 35.3% of the variance in malnutrition risk (Nagelkerke R^2^ = 0.353). As shown in Table 5, cardiac patients were significantly more likely to exhibit moderate/severe malnutrition compared to respiratory patients (OR = 9.07, 95% CI: 2.33–35.25, *p* = 0.001). Similarly, a lower Palliative Performance Scale (PPS < 50) was independently associated with a significantly increased risk of malnutrition (OR = 4.241, 95% CI: 1.204–14.940, *p* = 0.025). The absence of nutritional follow-up demonstrated a non-significant trend toward increased risk (OR = 0.419, 95% CI: 0.119–1.476, *p* = 0.176). In contrast, sex (female vs. male) and age (per year) were not significant predictors (OR = 1.471, 95% CI: 0.449–4.822, *p* = 0.524; and OR = 1.048, 95% CI: 0.960–1.145, *p* = 0.291, respectively).

#### 3.4.2. Ordinal Logistic Regression for Nutritional Severity (Three-Level CONUT)

To preserve the ordinal structure of the CONUT score and better reflect the continuum of nutritional risk, a secondary ordinal logistic regression was conducted using three categories: normal (0–1), mild (2–4), and moderate/severe (≥5) malnutrition (Table 6). The same covariates were included as in the binary model. The model showed good fit (χ^2^ = 28.14, *p* < 0.001; Nagelkerke R^2^ = 0.355), and the assumption of proportional odds was met (*p* = 0.954).

Cardiology remained the strongest independent predictor of worsening nutritional status (OR = 9.3; 95% CI: 2.9–30.3; *p* < 0.001). PPS < 50 was associated with greater odds of nutritional deterioration (OR = 3.65; 95% CI: 1.3–13.0, *p* = 0.014). Other covariates including age, sex, and nutritional follow-up were not statistically significant.

### 3.5. Mortality Analysis

Survival analysis using Kaplan–Meier estimates revealed no statistically significant differences in median survival times across clinical specialty, nutritional status, or follow-up intervention (Table 7).

Median survival was 177 days (95% CI: 28–326) for patients under cardiology disease (log-rank *p* = 0.797). Patients with moderate/severe malnutrition according to the dichotomized CONUT score (≥5) had a numerically shorter median survival of 99 days (95% CI: 0–214) compared to 224 days (95% CI: 157–291) for those classified as normal/mild (<5), though the difference was not statistically significant (*p* = 0.635). No statistically significant differences in survival were observed based on BMI categories, nutritional follow-up, or protein supplementation.

**Figure 1:**
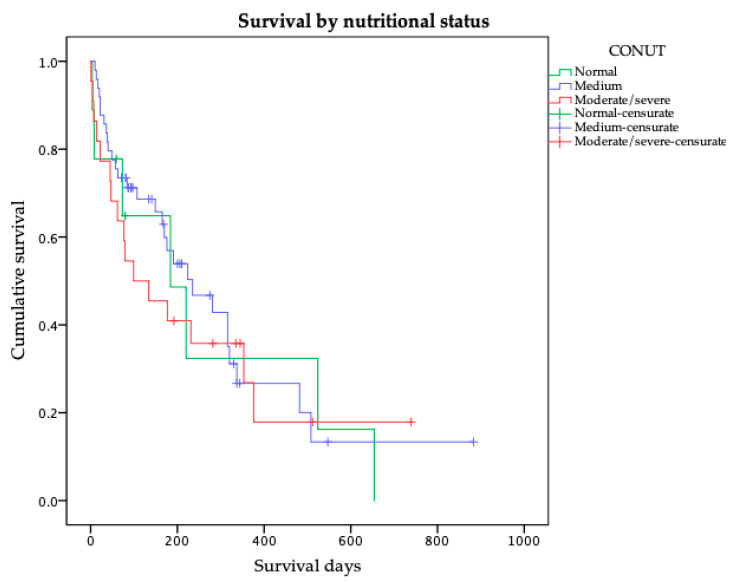
Kaplan–Meier Survival curves stratified by CONUT score categories (normal: 0–1, mild: 2–4, moderate/severe: ≥5). No significant differences were observed (log-rank *p* = 0.842).

Additionally, we analyzed survival using the three-level CONUT classification (normal, medium, and moderate/severe), consistent with the ordinal variable applied in the ordinal logistic regression. Although differences did not reach statistical significance (log-rank *p* = 0.842), Kaplan–Meier curves are presented in Figure 1 to visually illustrate the distribution of survival across these strata.

Cox regression analysis did not identify any statistically significant predictors of mortality among the evaluated variables (Table 8). Patients with medium or normal nutritional status, as assessed by the three-level CONUT score, did not show a significantly different hazard or death compared to those classified as moderate/severe (global *p* = 0.642). Similarly, follow-up by a clinical nutritionist (HR = 1.43, *p* = 0.254) and specialty (HR = 0.88, *p* = 0.704) were not associated with survival.

## 4. Discussion

This study explored the role of the CONUT score in identifying nutritional vulnerability among palliative patients with end-stage heart failure (HF) and chronic respiratory disease (CRD), highlighting disease-specific patterns and functional associations. This study revealed a significantly higher prevalence of moderate/severe malnutrition (CONUT ≥ 5) in HF patients. This nutritional vulnerability remained significant after adjusting for age, sex, and nutritional follow-up, suggesting an intrinsic link to disease trajectory and functional status, particularly in patients with low Palliative Performance Scale (PPS) scores. BMI was lower in HF patients, but it did not reliably reflect nutritional risk, unlike CONUT [24].

Malnutrition has been consistently reported in 30–50% of patients with advanced HF, frequently related to cardiac cachexia, sarcopenia, and systemic inflammation [25]. Our results are in line with the findings and highlight that even in the context of “preserved” or elevated BMI (due to edema), malnutrition may be present [26]. Although serum albumin has been widely described as a prognostic marker in patients with advanced heart failure, our findings challenge this association [27,28]. In our cohort, albumin levels were higher in cardiac patients despite a worse overall nutritional profile. This apparent paradox may be explained by the higher frequency of nutritional follow-up and protein supplementation observed in this group (49% vs. 36% in CRD), potentially leading to artificially elevated albumin values.

Moreover, in the palliative setting, albumin is strongly influenced by inflammation, fluid overload, hepatic congestion, and catabolic states, all of which may confound its interpretation as a marker of nutritional status or prognosis [27]. These results underscore the limited reliability of isolated biochemical markers in end-stage disease, where multifactorial processes often override traditional prognostic indicators.

In contrast, CRD patients showed better nutritional profiles, with lower rates of moderate/severe CONUT scores, consistent with literature suggesting malnutrition in 23% of advanced COPD and FPI cases [29,30]. Although CRD patients in our cohort showed relatively preserved biochemical nutritional markers, the high prevalence of exertional dyspnea suggests that functional limitations—possibly related to muscle wasting—persist despite adequate systemic indices. This aligns with previous findings linking malnutrition in COPD to reduced exercise tolerance and respiratory muscle weakness, highlighting the importance of integrating functional assessments into nutritional evaluation [31,32]. While previous studies have suggested a survival advantage in COPD patients with higher BMI—a phenomenon known as the “obesity paradox [33]—our data did not confirm this association in CRD patients. Despite a higher BMI in the respiratory group, no survival benefit was observed, and BMI did not correlate with nutritional risk or mortality. Although BMI correlated weakly and inversely with CONUT, the association was marginal. This finding highlights the limited clinical utility of BMI as a malnutrition marker in palliative patients, whereas CONUT captures immune and metabolic alterations beyond body wight. This absence may reflect the advanced palliative stage, where comorbidities and symptom burden outweigh anthropometric influences, or selection bias from the tertiary hospital setting, potentially skewing toward more severe cases. These findings reinforce the limited prognostic value of isolated anthropometric measures like BMI in end-stage disease and underscore the need for multifactorial assessment tools in palliative care populations.

Few studies have directly compared these two populations in palliative care, and our work contributes to this gap, suggesting that heart failure patients may face higher nutritional risk earlier in the disease trajectory.

Interestingly, we observed no significant association between nutritional status (CONUT), BMI, or protein supplementation and survival outcomes. This contrasts with previous studies in more stable HF or CRD population where malnutrition was a strong prognostic marker [34,35]. In our cohort, the advanced stage of illness, short follow-up, and multifactorial drivers of mortality (e.g., symptom burden, comorbidities, care goals) may have attenuated this association. The lack of significant survival differences by nutritional status may reflect the advanced disease stage, short follow-up (median 177–224 days), or multifactorial mortality drivers in palliative care. Future studies could explore time-dependent covariates or subgroup analyses (e.g., by HF subtype) to better elucidate nutritional impacts on survival.

Our findings suggest that nutritional markers such as CONUT are more valuable as indicators of physiological stress and quality of life needs than as independent predictors of mortality in end-of-life care.

The results highlight the clinical relevance of implementing nutritional screening in routine palliative care, especially in patients with heart failure [36]. While the higher prevalence of nutritional follow-up and protein supplementation in this group may reflect a greater perceived risk, our data do not suggest a survival benefit from these interventions. Rather, the clinical value of nutritional assessment lies in its capacity to guide symptom management, improve functional capacity, and support quality of life.

The use of CONUT in this population must be interpreted cautiously. Inflammatory processes, renal dysfunction, and fluid overload—common in palliative care—may artificially distort CONUT components like albumin and lymphocyte count. In fact, higher albumin levels observed in HF patients despite worse CONUT classification suggest possible confounding due to supplementation or volume status rather than true improvement. Therefore, CONUT may function more as a marker of physiological stress than a direct measure of nutritional intake or reserve [18,22].

Compared to GLIM criteria, which incorporate muscle mass and etiology [6], CONUT may overestimate malnutrition in HF due to low lymphocyte counts from systemic stress. The Prognostic Nutritional Index (PNI) [22] similarly includes albumin and lymphocytes but excludes cholesterol, potentially offering less sensitivity to lipid alterations in CRD. The higher albumin levels in HF patients despite worse CONUT scores may reflect frequent nutritional supplementation (49% in HF vs. 36% in CRD) or fluid retention masking true protein deficits [27]. Future studies should validate CONUT against muscle mass assessments (e.g., bioimpedance) to enhance specificity.

Limitations: The small sample size (N = 80) and limited events (22 for CONUT ≥ 5, 52 deaths) may have reduced statistical power, particularly for secondary outcomes like survival, resulting in wide confidence intervals and non-significant trends. Sensitivity analyses, such as bootstrapping, were not performed due to the limited sample size but could enhance robustness in future research. A small proportion of patients (n = 6) were undergoing renal replacement therapy, which may affect nutritional parameters and prognosis. However, the number was too low to allow meaningful stratified analysis, representing an additional limitation of our study. The cross-sectional design was based on a single time-point during palliative care evaluation. No longitudinal follow-up was conducted, and patients were at heterogeneous stages of disease trajectory, potentially affecting the interpretation of nutritional status and its associations. The single center setting limits generalizability. Missing data for some biochemical markers (e.g., prealbumin, CRP), and no objective measurements of muscle mass or function. Validated screening tools such as the Mini Nutritional Assessment (MNA) or the Nutritional Risk Score (NRS) were not systematically collected in our cohort, which limits the comparability of our results with other settings. This should be addressed in future studies. Although comparison with oncological palliative patients would provide valuable insights, our study was restricted to non-oncological populations. Future research should directly compare oncological and non-oncological cohorts to better contextualize nutritional vulnerability across palliative conditions. Finally, the CONUT score may be biased by inflammation, renal dysfunction, or acute decompensation, common in palliative population, thus limiting its specificity as a nutritional marker.

Future Directions: This study should be considered exploratory and larger, multicenter studies are needed to confirm these findings and explore longitudinal nutritional changes. Interventions trials targeting nutritional follow-up and protein supplementation in HF and CRD palliative care could clarify their impact on outcomes. Integrating CONUT into routine palliative assessment may facilitate timely and personalized care planning, particularly in identifying patients who could benefit most from tailored nutritional support.

## 5. Conclusions

Malnutrition in palliative care was significantly more prevalent among patients with end-stage heart failure than those with advanced respiratory disease. The CONUT score, despite its limitations, may serve as a pragmatic screening tool for identifying patients at nutritional and functional risk in advanced non-oncological palliative care. Its role appears more aligned with supporting clinical decision-making and personalized care planning than with predicting mortality.

## Figures and Tables

**Table 1:** Demographic, functional and mortality of the Study Population.

General Characteristics	All Patients (N = 80)	Cardiologic Disease (N = 41)	Respiratory Disease (N = 39)	*p* Value
Age (years), mean ± SD	77.8 ± 6.8	77.1 ± 7.6	78.5 ± 5.9	0.382
Sex (male), n (%)	52 (65%)	27 (66%)	25 (64%)	0.870
Origin, n (%)				0.751 *
Home	74 (92%)	38 (93%)	36 (92%)	
Nursing home	3 (4%)	2 (5%)	1 (3%)	
Intermediate Care Facility	3 (4%)	1 (2%)	2 (5%)	
Dementia, n (%)	4 (5%)	3 (7%)	1 (3%)	0.616 *
Dependence (Barthel Index), n (%)				0.925 *
Independence—100	23 (29%)	12 (29%)	11 (28%)	
Mild dependence—>60	42 (53%)	22 (54%)	20 (51%)	
Moderate dependence—40–55	7 (9%)	4 (10%)	3 (8%)	
Severe dependence—20–35	6 (7%)	2 (5%)	4 (10%)	
Total dependence—<20	2 (2%)	1 (2%)	1 (3%)	
Frailty—(CFS), n (%)				0.663 *
CFS 1	0 (0%)	0 (0%)	0 (0%)	
CFS 2	2 (2%)	2 (5%)	0 (0%)	
CFS 3	18 (23%)	10 (25%)	8 (21%)	
CFS 4	22 (28%)	9 (22%)	13 (33%)	
CFS 5	15 (17%)	7 (17%)	7 (18%)	
CFS 6	18 (23%)	9 (22%)	9 (23%)	
CFS 7	5 (6%)	3 (7%)	2 (5%)	
CFS 8	1 (1%)	1 (2%)	0 (0%)	
CFS 9	0 (0%)	0 (0%)	0 (0%)	
NECPAL, n (%)				**<0.001**
I (1–2)	11 (14%)	4 (10%)	7 (18%)	
II (3–4)	45 (56%)	16 (39%)	29 (74%)	
III (5–6)	24 (30%)	21 (51%)	3 (8%)	
PPS (Palliative Performance Scale), n (%)				0.490 *
>60	41 (51%)	22 (54%)	19 (49%)	
30–50	35 (44%)	16 (39%)	19 (49%)	
10–20	4 (5%)	3 (7%)	1 (3%)	
Mortality, n (%)	52 (65%)	30 (73%)	22 (56%)	0.116

*t*-test for independent samples. Chi-square test or * *p*-values for Fisher’s exact test (when ≥50% of cells had expected frequencies <5). *p*-values < 0.05 were considered statistically significant (in bold).

**Table 2:** Clinical and Symptom Characteristics of the Study Population.

Clinical and Symptoms Characteristics	All Patients (N = 80)	Cardiologic Disease (N = 41)	Respiratory Disease (N = 39)	*p* Value
**Cardiologic disease**				
Cardiac disease, n (%)				
Ischemic		8 (20%)		
Idiopathic/Familial Dilated		4 (10%)		
Hypertrophic		0 (0%)		
Valvular		15 (36%)		
Amyloidosis		2 (5%)		
Transplant		4 (10%)		
Ischemic-valvular		7 (17%)		
Others		1 (2%)		
LVEF, n (%)				
Normal ((50–55%)		11(27%)		
Mildly (41–49%)		5 (12%)		
Reduced ((40%)		8 (19%)		
Severely reduced (<30%)		17 (42%)		
NYHA, n (%)				
I		5 (12%)		
II		10 (24%)		
III		20 (49%)		
IV		6 (15%)		
Cardiac treatment, n (%)				
Inotropes (Levosimendan or Dobutamine)		10 (25%)		
Hemodialysis (Peritoneal or conventional)		3 (7%)		
Inotropes + Peritoneal Dialysis		3 (7%)		
Diuretic		23 (56%)		
External ventricular drain		2 (5%)		
**Respiratory disease**				
Respiratory disease, n (%)				
Chronic Obstructive Pulmonary Disease			15 (38%)	
Diffuse Interstitial Lung Disease			19 (49%)	
Pulmonary Hypertension			2 (5%)	
Other			3 (8%)	
mMRC, n (%)				
1			0 (0%)	
2			2 (5%)	
3			32 (82%)	
4			5 (13%)	
Respiratory treatment, n (%)				
Home oxygen			29 (%)	
Home high-flow nasal cannula			1 (%)	
Nocturnal CPAP			2 (%)	
Bronchodilator therapy			6 (%)	
Other			1 (%)	
**Symptoms**				
Dyspnea, n (%)				**<0.001**
At rest	20 (25%)	7 (17%)	13 (33%)	
On effort	38 (48%)	13 (32%)	25 (64%)	
No dyspnea	22 (27%)	21 (51%)	1 (3%)	
Anorexia, n (%)	38 (48%)	24 (59%)	14 (36%)	**0.043**
Asthenia/Fatigue, n (%)	44 (55%)	24 (58%)	20 (51%)	0.514
Xerostomia, n (%)	35 (44%)	14 (34%)	21 (54%)	0.076
Dysphagia, n (%)	9 (11%)	6 (15%)	3 (8%)	0.483 *
Pain, n (%)	15 (19%)	6 (15%)	9 (23%)	0.334
Constipation, n (%)	23 (29%)	13 (32%)	10 (26%)	0.549

*t*-test for independent samples. Chi-square test or * *p*-values for Fisher’s exact test (when ≥50% of cells had expected frequencies <5). *p*-values < 0.05 were considered statistically significant (in bold).

**Table 3:** Nutritional and Biochemical Characteristics of the Study Population.

Nutritional Characteristics		All Patients (N = 80)	Cardiologic Disease (N = 41)	Respiratory Disease (N = 39)	*p* Value
Weight loss in the last 6 months, n (%)		18 (23%)	11 (27%)	7 (18%)	0.342
Nutritional follow-up, n (%)		44 (55%)	27 (66%)	17 (44%)	**0.045**
Protein supplementation, n (%)		34 (42%)	20 (49%)	14 (36%)	0.244
High adherence, n (%)		31 (91%)	14 (100%)	17 (85%)	0.251 *
BMI(Kg/m^2^), median (IQR)		23.56 (5.36)	22.65 (5.36)	25.13 (I8.87)	**0.045**
BMI classification, n (%)					0.052
Underweight—(<18.5)		10 (13%)	5 (12%)	5 (13%)	
Normal weight—(18.5–24.9)		39 (49%)	25 (61%)	14 (36%)	
Overweight—(25.0–29.9)		20 (25%)	9 (22%)	11 (28%)	
Obesity (>30.0)		11 (13%)	2 (5%)	9 (24%)	
CONUT score, n (%)					0.002 *
Normal (0–1)		9 (11%)	1 (2%)	8 (21%)	
Mild (2–4)		49 (61%)	22 (54%)	27 (69%)	
Moderate (5–8)		19 (24%)	15 (37%)	4 (10%)	
Severe (9–12)		3 (4%)	3 (7%)	0 (0%)	
Biochemical characteristics	N				
Total proteins (g/L), mean ± SD	80	62.83 ± 8.9	65.98 ± 8.85	59.51 ± 7.77	**0.001**
Albumin (g/L), median (IQR)	80	38.00 (5)	39.00 (6)	36.00 (6)	0.090
Prealbumin (g/L), mean (SD)	45	0.18 (0.07)	0.21 (0.07)	0.15 (0.07)	**0.006**
Total cholesterol mg/dL, median (IQR)	80	156.00 (76)	125.00 (59)	175.00 (68)	**0.000**
Triglycerides (mg/dL), median (IQR)	80	101.5 (56)	101.00 (55)	107.00 (48)	0.461
Phosphorus (mg/dL), median (IQR)	64	3.45 (1.2)	3.75 (1.1)	3.20 (0.6)	**0.005**
Magnesium (mg/dL), mean (SD)	77	2.05 (0.31)	2.10 (0.38)	1.99 (0.22)	0.118
Zinc (µg/dL), median (IQR)	31	71.45 (14.21)	68.00 (12.23)	73.94 (15.33)	0.257
Calcium (mg/dL), median (IQR)	78	8.70 (0.5)	9.00 (0.8)	8.60 (0.5)	**0.019**
Creatinine (mg/dL), median (IQR)	80	1.15 (1.41)	2.05 (1.75)	0.81 (0.25)	**0.001**
Lymphocyte (×10^9^/L), median (IQR)	80	1.20 (1.00)	0.82 (0.80)	1.70 (1.10)	**0.000**
PCR (mg/dL), median (IQR)	72	1.05 (2.77)	1.07 (1.46)	0.40 (5.50)	0.312

*t*-test for independent samples. Mann–Whitney U test. Percentages are calculated within each specialty (column percentages). All variables were analyzed using 2 × 2 contingency tables. * *p*-values from Fisher’s exact test due to expected frequencies <5 in ≥50% of cells. A *p*-value < 0.05 indicates a statistically significant association (in bold). The number of available cases (N) is indicated per variable due to missing values.

**Table 4:** Univariate Analysis of Factors Associated with Nutritional Status (CONUT Dichotomized: Normal/Mild vs. Moderate/Severe).

Variable	Normal/Mild (N = 58)	Moderate/Severe (N = 22)	OR (95% CI)	*p*-Value
Demographics				
Sex, male, n (%)	39 (67%)	13 (51%)	Ref.	0.601 *
Female, n (%)	19 (33%)	9 (41%)	1.42 (0.51–3.94)	
Age, median (IQR)	78.0 (8.0)	78.5 (9.0)	0.99 (0.93–1.06)	0.871 **
Clinical Variables, n (%)				0.001 *
Respiratory	35 (60%)	4 (18%)	Ref.	
Specialty, Cardiology	23 (40%)	18 (82%)	6.85 (2.06–22.78)	
NECPAL, n (%)				0.013 *
NECPAL Stage I	9 (15%)	2 (9%)	0.22 (0.04–1.26)	
NECPAL Stage II	37 (64%)	8 (36%)	0.22 (0.07–0.66)	
NECPAL Stage III	12 (21%)	12 (55%)	Ref.	
PPS, n (%)				0.001 *
PPS >60	34 (59%)	7 (32%)	Ref.	
PPS 30–50	24 (41%)	11 (50%)	2.23 (0.75–6.53)	
PPS 10–20	0 (0%)	4 (18%)	Not calculable ^‡^	
Barthel, n (%)				0.007 *
Barthel: Independent	21 (36%)	2 (9%)	Ref.	
Barthel: Mild dependence	26 (45%)	16 (73%)	6.46 (1.33–31.37)	
Barthel: Moderate/Severe	11 (19%)	4 (18%)	3.82 (0.62–23.52)	
LVEF, n (%)				0.642 *
Normal (≥50–55)	5 (22%)	6 (33%)	Ref.	
Mildly (41–49%)	2 (9%)	3 (17%)	1.25 (0.15–10.44)	
Reduced (≤40%)	5 (22%)	3 (17%)	0.50 (0.08–3.20)	
Severely reduced (<30%)	11 (48%)	6 (33%)	0.45 (0.10–2.11)	
Symptoms				
Pain (Yes)	43 (74%)	22 (100%)	Not calculable ^‡^	0.008 *
Dysphagia (Yes)	6 (10%)	3 (14%)	1.37 (0.31–6.03)	0.700 *
Anorexia (Yes)	24 (41%)	14 (64%)	2.48 (0.89–6.91)	0.086 *
Xerostomy (Yes)	24 (41%)	11 (50%)	1.42 (0.52–3.85)	0.615 *
BMI classification, n (%)				0.403 *
Underweight—(<18.5)	6 (10%)	4 (18%)	6.67 (0.60–74.34)	
Normal weight—(18.5–24.9)	27 (47%)	12 (54%)	4.44 (0.51–38.74)	
Overweight—(25.0–29.9)	15 (26%)	5 (23%)	3.33 (0.34–32.83)	
Obesity (>30.0)	10 (17%)	1 (5%)	Ref.	
Nutritional characteristics, n (%)				
Weight loss in the last 6 month	14 (24%)	4 (18%)	0.70 (0.20–2.42)	0.766 *
Nutritional follow-up (Yes)	27 (47%)	17 (77%)	3.91 (1.29–11.86)	0.022 *
Protein supplementation (Yes)	21 (36%)	13 (60%)	2.55 (0.93–6.96)	0.080 *
Biochemical characteristics				
Albumin, g/L (median)	39.0 (5.0)	30.5 (7.0)	0.85 (0.77–0.94) ^†^	0.004 *
Total cholesterol, mg/dL	165.0 (72.0)	119.0 (37.0)	0.98 (0.97–0.99) ^†^	0.001 *
Total protein, g/L	64.0 (10.0)	58.0 (12.0)	0.94 (0.89–0.99) ^†^	0.029 *
Creatinine, mg/dL	0.98 (1.10)	1.42 (1.80)	1.36 (0.99–1.87) ^†^	0.047 *
Lymphocytes, (×10^9^/L)	1.3 (1.0)	0.9 (0.8)	0.54 (0.31–0.93) ^†^	0.002 *

OR = Odds Ratio; CI = Confidence Interval; Ref = Reference category. *p*-values from Chi-square or Fisher’s exact test (*); Mann–Whitney U test (**). ^†^ OR estimated by univariate logistic regression (per unit change unless § per 10 mg/dL cholesterol). ^‡^ OR not calculated due to zero-cell counts (only proportion difference reported). Sample size: N = 80 for all variables except prealbumin (N = 45) and CRP (N = 26), excluded due to missing data. A *p*-value < 0.05 indicates a statistically significant association (in bold).

**Table 5:** Multivariate Logistic Regression Analysis of Predictors of Moderate/Severe Malnutrition (CONUT ≥ 5).

Predictor	OR (Exp(B))	95% CI	*p*-Value
Specialty (Cardiology vs. Pulmonology)	9.07	2.33–35.25	**0.001**
PPS (<50 vs. >60)	4.24	1.20–14.94	**0.025**
Nutritional follow-up (No vs. Yes)	0.42	0.12–1.48	0.176
Sex (Female vs. Male)	1.47	0.45–4.82	0.524
Age (per year)	1.05	0.96–1.15	0.291

Hosmer–Lemeshow *p* = 0.903; Nagelkerke R^2^ = 0.353. A *p*-value < 0.05 indicates a statistically significant association (in bold).

**Table 6:** Ordinal Logistic Regression for Nutritional Severity Based on CONUT Score.

Predictor	OR (Exp(B))	95% CI	*p*-Value
Specialty (Cardiology vs. Pulmonology)	9.30	2.90–30.30	**<0.001**
PPS (<50 vs. >60)	3.65	1.30–13.00	**0.014**
Age (per year increase)	1.06	0.99–1.14	0.100
Nutritional follow-up (No vs. Yes)	0.50	0.18–1.42	0.196
Sex (Female vs. Male)	1.32	0.51–3.58	0.580

Ordinal logistic regression model with proportional odds assumption met (Test of Parallel Lines: χ^2^ = 1.10, *df* = 5, *p* = 0.954). Model fit: χ^2^ = 28.14 (*df* = 5, *p* < 0.001); Nagelkerke R^2^ = 0.355; Pearson χ^2^ = 115.75 (*p* = 0.792); Deviance = 97.30 (*p* = 0.983). A *p*-value <0.05 indicates a statistically significant association (in bold).

**Table 7:** Median Survival Times (Kaplan–Meier) by Clinical and Nutritional Variables.

Variable	Group	Median Survival (Days)	95% CI	Log-Rank *p*-Value
Specialty	Cardiology	177	28–326	0.797
	Pulmonology	220	146–294	
CONUT (Score)	Normal/Mild (<5)	224	157–291	0.635
	Moderate/Severe (≥5)	99	0–214	
Nutritional Status (BMI)	Undernutrition	170	133–207	0.590
	Normal weight	177	27–327	
	Overweight	320	119–521	
	Obesity	235	7–463	
Nutritional Follow-up	No	191	78–304	0.412
	Yes	231	91–371	
Protein Supplements	No	220	159–281	0.882
	Yes	177	78–276	

Kaplan–Meier analysis. N = 80; Events = 52; Censored = 28.

**Table 8:** Cox regression for Mortality Predictors.

Predictor	HR (Exp(B))	95% CI for HR	*p*-Value
Nutritional follow up (No vs. Yes)	1.430	0.757–2.699	0.254
Specialty (Cardiology vs. Respiratory)	0.878	0.447–1.725	0.704
CONUT–Normal vs. Moderate/Severe	0.978	0.334–2.862	0.967
CONUT—Mild vs. Moderate/Severe	0.747	0.376–1.486	0.409

Cox regression model. Reference category for CONUT: Moderate/Severe. Model *χ^2^* = 1.709, *df* = 4, *p* = 0.789.

## Data Availability

The data presented in this study are available on request from the corresponding author. The data are not publicly available due to the ethical and privacy restrictions.

## Abbreviations

The following abbreviations are used in this manuscript:

BMI	Body Mass Index
Barthel	Barthel Index
CONUT	Controlling Nutritional Status
COPD	Chronic Obstructive Pulmonary Disease
CFS	Clinical Frailty Scale
CRD	Chronic Respiratory Disease
CPAP	continuous positive airway pressure
CI	confidence interval
CRP	C-reactive protein
HF	Heart Failure
ILD	Diffuse Interstitial Lung Disease
IQR	interquartile range
LVEF	Left Ventricular Ejection Fraction
mMRC	Modified Medical Research Council
NECPAL	Necesidades Paliativas (Palliative Care Needs)
NYHA	New York Heart Association
OR	odds ratio
PH	Pulmonary Hypertension
PPS	Palliative Performance Scale
Ref.	reference category
SD	standard deviation
